# Chronic non-transmural infarction has a delayed recovery of function following revascularization

**DOI:** 10.1186/1471-2261-10-4

**Published:** 2010-01-18

**Authors:** Martin Ugander, Peter A Cain, Per Johnsson, John Palmer, Håkan Arheden

**Affiliations:** 1 Department of Clinical Physiology, Lund University Hospital, Lund University, Lund, SE-221 85 Sweden; 2 Department of Cardiothoracic Surgery, Lund University Hospital, Lund University, Lund, SE-221 85 Sweden; 3 Department of Medical Radiation Physics, Lund University Hospital, Lund University, Lund, SE-221 85 Sweden

## Abstract

**Background:**

The time course of regional functional recovery following revascularization with regards to the presence or absence of infarction is poorly known. We studied the effect of the presence of chronic non-transmural infarction on the time course of recovery of myocardial perfusion and function after elective revascularization.

**Methods:**

Eighteen patients (mean age 69, range 52-84, 17 men) prospectively underwent cine magnetic resonance imaging (MRI), delayed contrast enhanced MRI and rest/stress 99m-Tc-tetrofosmin single photon emission computed tomography (SPECT) before, one and six months after elective coronary artery bypass grafting (CABG) or percutaneous coronary intervention (PCI).

**Results:**

Dysfunctional myocardial segments (n = 337/864, 39%) were classified according to the presence (n = 164) or absence (n = 173) of infarction. Infarct transmurality in dysfunctional segments was largely non-transmural (transmurality = 31 ± 22%). Quantitative stress perfusion and wall thickening increased at one month in dysfunctional segments without infarction (p < 0.001), with no further improvement at six months. Despite improvements in stress perfusion at one month (p < 0.001), non-transmural infarction displayed a slower and lesser improvement in wall thickening at one (p < 0.05) and six months (p < 0.001).

**Conclusions:**

Dysfunctional segments without infarction represent repetitively stunned or hibernating myocardium, and these segments improved both perfusion and function within one month after revascularization with no improvement thereafter. Although dysfunctional segments with non-transmural infarction improved in perfusion at one month, functional recovery was mostly seen between one and six months, possibly reflecting a more severe ischemic burden. These findings may be of value in the clinical assessment of regional functional recovery in the time period after revascularization.

## Background

Revascularization of dysfunctional but viable myocardium in patients with chronic ischemic heart disease (CIHD) may offer both functional improvement of myocardium and prognostic benefit [1]. The pathophysiology underlying the development and recovery of hypofunctioning but viable myocardium in CIHD is not completely understood [2]. Myocardium which is hypofunctioning but viable at rest may represent either hibernating or repetitively stunned myocardium [2]. Therefore, for the purposes of this article, we will use the collective term "dysfunctional but viable myocardium". Dysfunctional myocardial segments have been shown to improve function immediately post-operatively with no further change 8 days after CABG [3]. This finding indicates that functional recovery begins early. However, others have shown that continued functional recovery is present at follow-up between three and 14 months later [4-9]. Thus, previous studies have shown varying results with regards to the time course of functional recovery following revascularization for dysfunctional segments with or without infarction, and remote myocardium, respectively.

Delayed contrast enhanced magnetic resonance imaging (DE-MRI) has shown to be valuable for predicting regional functional improvement after revascularization [10]. Furthermore, perfusion of dysfunctional but viable myocardium has been shown to improve soon after revascularization [11]. However, it is not known if the time course of recovery for perfusion and function following revascularization is the same for dysfunctional segments with or without the presence of non-transmural infarction as determined by DE-MRI. Therefore, we sought to quantitatively assess the influence of the presence of non-transmural myocardial infarction on the time course for regional recovery of function by MRI, and perfusion by ^99m^Tc-tetrofosmin single photon emission computed tomography (SPECT) over a six month period after revascularization.

## Methods

### Study population

**Figure 1:**
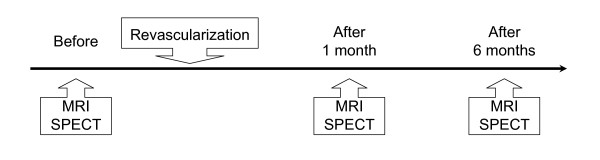
**Timeline of events in the study**. Patients were imaged with both MRI and rest/stress SPECT before revascularization, and after both one and six months.

The study was approved by the ethics committee on human research at Lund University Hospital. All patients provided written informed consent. Patients were prospectively enrolled between December, 2001 and May, 2005. The inclusion criterion was clinical selection for first time elective revascularization by coronary artery bypass grafting (CABG) or percutaneous coronary intervention (PCI). Following revascularization, changes in medication were determined by the caring physician. Patients were imaged with cardiac MRI and rest/stress SPECT prior to revascularization and one and six months after revascularization. A schematic diagram of the timeline of the study is presented in Figure 1. Exclusion criteria were valvular surgery in adjunct to revascularization, acute coronary syndrome during the course of the study, New York Heart Association functional class IV, absence of sinus rhythm, claustrophobia or contraindications for MRI.

### MR Imaging

Left ventricular function and viability were imaged in the short-axis plane during breath hold using a 1.5T system (Magnetom Vision, Siemens, Erlangen, Germany or Intera CV, Philips, Best, the Netherlands). Global and regional function was imaged using an ECG-triggered cine gradient echo sequence (Siemens, resolution 1.6 × 1.6 × 8 mm, gap 2 mm, temporal resolution 50 ms) or a retrospectively vector-ECG-triggered cine steady-state free precession sequence (Philips, spatial resolution 1.25 × 1.25 × 8 mm, gap 0 mm, temporal resolution 33 ms, SENSE factor 2). Infarct imaging was undertaken using a segmented inversion recovery turbo fast low-angle shot sequence [12] in either 2D or 3D (2D: Siemens, acquisition every other heart beat, resolution 1.6 × 1.6 × 8 mm, gap 2 mm, 3D: Philips, acquisition every heart beat, 1.6 × 1.6 × 8 mm, gap 0 mm, inversion time set to null normal myocardium). Imaging was performed 15-20 minutes after intravenous injection of 0.2 mmol/kg body weight of an extracellular contrast agent (Magnevist^®^, gadopentetate dimeglumine, Gd-DTPA, Schering Nordiska AB, Järfälla, Sweden). This approach has been shown to enhance non-viable myocardium [13] due to an increased tissue distribution volume of Gd-DTPA in non-viable regions [14].

### SPECT Imaging

Rest and stress SPECT imaging were performed on separate days, 30 minutes after intravenous injection of a body weight adjusted dose (500-700 MBq) of ^99m^Tc-tetrofosmin (Amersham Health, Buckinghamshire, UK). The same dose was used for rest and stress. For stress imaging, 5 mg/ml adenosin (Adenosin Item^®^, Item Development AB, Stocksund, Sweden) was infused at a rate of 140 μg/kg/min for 3 minutes before tracer injection, and continued for 2 minutes following injection. For those instances where a subject mistakenly had consumed caffeine in the 24 hours prior to stress imaging, exercise stress was performed using a bicycle ergometer with a minimum required increase of 85% of the maximum predicted heart rate. For both rest and stress imaging, the subject was imaged in the supine position with a dual head camera (ADAC Vertex, Milpitas, California, USA) using standard methods [15].

### Image Analysis

**Figure 2:**
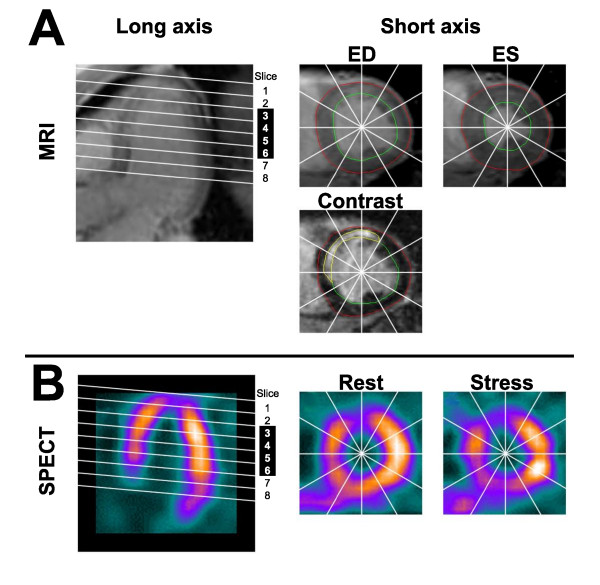
**Methods for quantitative assessment of function, infarction and perfusion**. **(A) **A long axis magnetic resonance image (MRI) was used to plan short axis images of the left ventricle (LV) (parallel white lines). Slices 3-6 were used for analysis. Endocardial and epicardial borders of the LV were manually delineated on short axis cine MR images in end diastole (ED) and end systole (ES). Wall thickness was quantified in a 12-segment model. Delayed enhancement images (Contrast) were obtained at the same slice positions. Transmurality of infarction was quantified using manual delineation of the borders of the LV endocardium, epicardium and infarction, employing the same 12-segment model. **(B) **Single photon emission computed tomography (SPECT) images were reconstructed in the same fashion as for MRI. Regional perfusion at rest and stress were assessed quantitatively using the same 12-segment model. See text for details.

All studies were quantitatively analyzed using a 48-segment model with 4 midventricular short-axis slices and 12 segments per slice. All images were analyzed using software developed in-house, including the freely available software Segment version 1.661 http://segment.heiberg.se [16], as described previously in detail [17]. In summary, the endocardial and epicardial borders of short axis cine MR images were manually delineated in end diastole and end systole for determination of wall thickening. The endocardial and epicardial borders, and the border of the hyperenhanced region of infarction in DE-MRI were manually delineated for determination of infarct transmurality. Infarct transmurality was defined as the transmural extent of infarction divided by the total transmural wall thickness. Semi-automatic quantification of wall thickening from MRI, infarction from DE-MRI, and both rest and stress perfusion from SPECT was performed along radial profiles from the centroid of the left ventricular lumen at every other degree, yielding 180 measurements per short axis slice. For any given parameter, measurements were averaged over 30 degrees to obtain values for the 12-segment per slice model. The four slices analyzed for all subjects were either 3.0, 4.0, 5.0 and 6.0 cm from the apex (Siemens) or 3.2, 4.0, 4.8 and 5.6 cm (Philips) from the apex, respectively. Slices closest to the base and apex were excluded to minimize errors in assessment of wall thickness introduced by the partial volume effect and atrioventricular plane movement, respectively. Dysfunction was defined as segments with wall thickening less than 30% [18]. For MR images, the apex was defined at the time of imaging by commencing the short axis imaging with an apical slice that encompassed the apical tip of the heart. For SPECT, the apex was defined manually in a vertical long axis image, after which contiguous short axis slices were reconstructed. Slice thickness for reconstructed images were 10 mm or 8 mm for patients imaged by MR using Siemens or Philips scanners, respectively. See Figure 2.

### Statistics

Time course data are presented as mean ± SEM. The number of days between imaging and revascularization are presented as median and interquartile range (IQR), defined as the range between the 25^th ^and 75^th ^quartile of the data. All other data are presented as mean ± SD unless stated otherwise. Variations between data were tested by paired t-test and independent t-test as appropriate. P < 0.05 was considered statistically significant. Power analysis was calculated retrospectively to determine the difference in wall thickening which one could detect with a power of 0.90 using p < 0.05 (two-tailed), a measurement variability (SD) of 1 mm, and given the number of myocardial segments involved in the comparison.

## Results

Patient characteristics including times of imaging in relation to revascularization and baseline medications are described in Table 1. LVEF was unchanged one month and at six months following revascularization (44 ± 12%, p = 0.253 and 47 ± 13%, p = 0.225). Upon discharge following revascularization, all patients' medications were unchanged or dosages had been reduced except for the following. One patient had an increase in the dosage of both beta-blockers and diuretics. Five separate patients had either an increase in beta-blockers, angiotensin converting enzyme inhibitors or diuretics, respectively. No patient experienced perioperative infarction or died during the study. Two patients were included but lost to follow-up, and therefore not included in the analysis.

**Table 1:** Patient characteristics

Mean age, y	69 (range 53-84)
Male, n (%)	17 (94)
Caucasian race, n (%)	18 (100)
1-vessel disease, n (%)	3 (17)
2-vessel disease, n (%)	8 (44)
3-vessel disease, n (%)	7 (39)
Diabetes, n (%)	7 (39)
Baseline LVEF*, %	47 ± 11
Baseline medication with...	
Beta-blocker, n (%)	15 (83)
Angiotensin converting enzyme inhibitor, n (%)	10 (56)
Diuretic, n (%)	5 (28)
Baseline MR, median days from revascularization	-4 (IQR -7 to -2)
Baseline rest SPECT, median days from revascularization	-4 (IQR -7 to -4)
Baseline stress SPECT, median days from revascularization	-1 (IQR -6 to -1)
One month MR, median days from revascularization	35 (IQR 29 to 42)
One month SPECT, median days from revascularization	31 (IQR 28 to 38)
One month SPECT, median days from revascularization	33 (IQR 29 to 41)
Six month MR, median days from revascularization	196 (IQR 187 to 207)
Six month SPECT, median days from revascularization	196 (IQR 187 to 207)
Six month SPECT, median days from revascularization	197 (IQR 188 to 208)

* denotes that LVEF data was not available for one patient due to insufficient number of MR images. IQR = interquartile range.

### Delayed enhancement imaging prior to CABG

**Figure 3:**
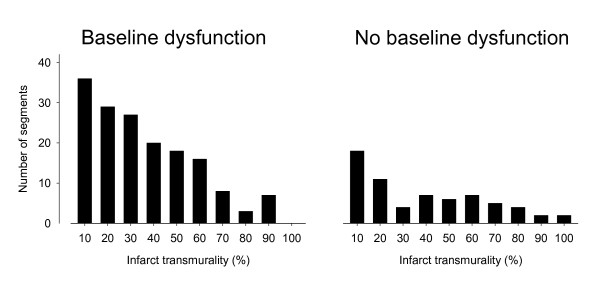
**The distribution of infarct transmuralities in the population**. Data are presented according the presence (left) or absence (right) of dysfunction (wall thickening <30%) prior to revascularization. Note the predominantly non-transmural distribution of the infarct transmuralities.

DE-MRI was possible in all patients resulting in 864 segments for analysis. Myocardial segments that were dysfunctional before revascularization (n = 337, 39%) were classified according to the presence (n = 164) or absence (n = 173) of infarction. Figure 3 shows the distribution of infarct transmuralities for segments that were dysfunctional and segments that were not dysfunctional prior to revascularization. Among dysfunctional segments with infarction, infarct transmurality was 31 ± 22%, range 1-87%.

### Time course of regional function

**Figure 4:**
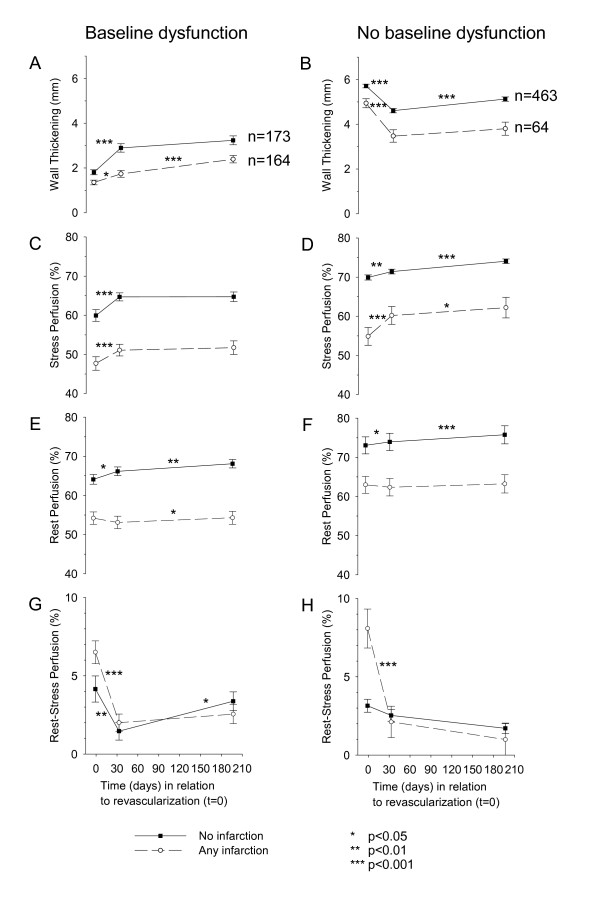
**Function and perfusion after revascularization for segments according to presence of dysfunction before revascularization**. Quantitative wall thickening (A, B) was assessed by cine magnetic resonance imaging (MRI). Stress perfusion (C, D) and rest perfusion (E, F) were assessed by ^99m^Tc-tetrofosmin single photon emission computed tomography (SPECT). Reversible perfusion is presented as the difference between rest and stress perfusion (G, H). Black squares and open circles denote myocardial segments without and with infarction, respectively, as assessed by delayed enhancement MRI. Error bars represent SEM.

Retrospective power analysis showed that given a comparison of 164 myocardial segments, there was a power of 0.90 for detecting a change in wall thickening of 0.25 mm. Figure 4A shows the time course of regional wall thickening for segments that were dysfunctional before surgery. Non-infarcted segments increased in function at one month (p < 0.001) with no change thereafter (p = 0.104). Segments with infarction, however, showed improvement in function at one month (p = 0.013), but also improvement in function between one and six months (p < 0.001). Figure 4B shows that segments with no baseline dysfunction showed a decrease in wall thickening at one month regardless of presence of infarction (p < 0.001). Among these segments, only those without infarction showed a significant increase in function between one and six months (p < 0.001).

### Time course of regional perfusion

#### Stress Perfusion

Figure 4C shows the time course of quantitative stress perfusion for segments that were dysfunctional before surgery. Segments with and without infarction differed from each other in stress perfusion at all time points (p < 0.001). Both groups showed a similar time course of improvement at one month (p < 0.001) and no further improvement at six months (p = 0.366 and p = 0.965). Figure 4D shows that in segments with no baseline dysfunction both with and without infarction, stress perfusion increased mostly at one month (p < 0.001 and p = 0.002), but also a six months (p = 0.042 and p < 0.001).

#### Rest Perfusion

Figure 4E shows the time course of rest perfusion for segments that were dysfunctional before surgery. Both groups differed in rest perfusion from each other at all time points (p < 0.001). Only non-infarcted segments improved in resting perfusion between baseline and one month (p = 0.010) and six months (p = 0.002). Figure 4F shows that in segments with no baseline dysfunction and without infarction, rest perfusion increased at both one month (p = 0.023) and six months (p < 0.001), while segments with infarction remained unchanged at both time points (p = 0.539 and p = 0.348).

#### Perfusion difference

Figure 4G shows the time course of the quantitative difference between rest and stress perfusion for segments that were dysfunctional before surgery. The groups only differed significantly from each other at baseline (p = 0.035). Both segments with and without infarction decreased significantly between baseline and one month (p = 0.008 and p < 0.001) with only non-infarcted segments showing a change between one and six months (p = 0.025). Figure 4H shows that among segments with no baseline dysfunction, only those with infarction were relieved of their perfusion difference at one month (p = 0.001).

## Discussion

To our knowledge, this is the first study to have serially and quantitatively examined the time course after revascularization of regional recovery of perfusion and function according to presence of infarction by DE-MRI. The major findings can be summarized as follows. Dysfunctional segments without infarction gained the greatest improvement in both perfusion and function within one month after revascularization. In contrast, segments with infarction, which was predominantly <50% transmural, showed a slower functional recovery which was obvious first at six months, despite improvements in perfusion already at one month.

### Time course of recovery of regional function

Several studies have examined functional recovery at single time points following revascularization based on the extent of infarction assessed by DE-MRI [10,19,20]. Studies using echocardiography and scintigraphic techniques have demonstrated that the time course of regional functional recovery following CABG begins immediately following revascularization and may continue up to between three and 14 months after surgery [3-9]. For example, one study showed no functional improvement in segments with either non-transmural or transmural infarction as determined by ^18^F-2-deoxyglucose (FDG) uptake using SPECT [8]. Yet, segments with reduced perfusion but no sign of infarction using FDG improved function both three and 14 months after revascularization. The discrepancy in the time course of functional recovery between previous results and ours may be due to the limited spatial resolution of SPECT for defining the presence and transmurality of infarction in previous studies, while we used DE-MRI. Similar to our findings, it was recently shown that the time course of regional functional recovery following revascularization increased with the amount of regional infarction as determined by DE-MRI [21]. However, that study did not assess perfusion, as in the current study.

Late improvements in function assessed visually by echocardiography have been associated with pre-operative histological and scintigraphic measures of ischemic burden, defined as a prolonged duration, greater extent or greater severity of ischemia [4-9]. Such histological measures of ischemic burden have been shown to independently correlate to both scintigraphic findings [22] and an increased duration of ischemia resulting in a delayed functional recovery [23]. Therefore, one potential explanation for our findings of the difference in time course of functional recovery between segments with and without infarction may be a more severe degree of ischemic burden in the viable portion of the segments with infarction. This is supported by our finding of a greater initial perfusion difference in these segments. Alternatively, changes in afterload or compensatory hypertrophy of viable myocardium following revascularization may have contributed to improved regional function, but this was not studied.

Surprisingly, segments without baseline dysfunction showed a reduction in wall thickening at one month regardless of presence of infarction. The reason for this pattern of remodelling is not known. Although we found changes in regional function in different regions within the left ventricle, we found no change in net global function (ejection fraction) following revascularization in our population. Similar to our findings, stent revascularization of chronic total coronary occlusion has been shown to increase wall thickening in dysfunctional segments, but decrease wall thickening in remote segments, with no net change in ejection fraction [24]. Also, ventriculography has been used to report improved regional wall motion in hypokinetic segments but also simultaneous deterioration in wall motion in initially normokinetic segments [25]. Our findings of simultaneous improvement and deterioration of regional function in segments of different baseline functional status seems reasonable considering the lack of change in LVEF in our population. Thus, the improved outcome in revascularized patients [1], despite sometimes limited improvements in ejection fraction, may be caused not only by a relief of ischemia in ischemic but viable regions but also a relief of compensatory hyperfunction in remote and normally functioning myocardium.

### Time course of recovery of regional perfusion

#### Rest Perfusion

The current study agrees with previous findings that scintigraphically assessed regional perfusion at rest improves soon after surgical and percutaneous revascularization [11].

#### Stress Perfusion

The current study also agrees with previous findings showing an improvement in scintigraphically assessed stress perfusion at single examinations after revascularization. This has been shown soon after percutaneous revascularization [26] and both 2-5 weeks [4] and 3-4 months [27] after surgical revascularization. Manyari *et al *[28], showed a progressive improvement in stress perfusion at 9 days and 3 months, but not 6 months following percutaneous revascularization. Taken together with our findings, it appears that incremental improvement in perfusion can be identified no later than 3 months after revascularization.

#### Perfusion difference

The difference between rest and stress perfusion may represent stress induced ischemia. Previous studies have examined the extent of reversible perfusion defects that exceed a certain severity cut-off value [29]. To our knowledge, this is the first study to examine the severity of reversibility of perfusion for given segments in this manner. Our results for both perfusion and function are consistent with the proposed mechanism that a greater ischemic burden may be involved in the delayed functional recovery of segments with infarction.

### Infarct transmurality and baseline function

When comparing the distribution of infarct transmurality in relation to baseline dysfunction, the current study showed, as expected, that there was a greater prevalence of infarction among segments with baseline dysfunction. The distribution of infarction in the population was largely non-transmural. However, it is noteworthy that there existed segments without baseline dysfunction which had as much as 100% infarct transmurality. This may seem counterintuitive. However, this represents very few myocardial segments (n = 2), and can be explained by tethering to adjacent segments. For example, it has been shown that myocardial segmental function is influenced moreso by the function of neighbouring segments than by infarct transmurality as such [30].

### Limitations of the Study

The number of patients is limited, however, power analysis showed that the study was adequately powered to assess differences in regional function. The number of segments with different degrees of transmurality did not permit an adequate separate analysis for infarction of different transmuralities. Although care was taken to achieve maximal alignment of slices between modalities and time points, misalignment due to fundamental differences in the imaging modalities is also a potential limitation.

## Conclusions

This study has demonstrated that dysfunctional segments without infarction, also known as hibernating or repetitively stunned myocardium, improved in both perfusion and function within one month after revascularization. Although segments with predominantly non-transmural infarction improved in rest and stress perfusion at one month, functional recovery was slower and clearly seen first at six months. Despite lack of change in ejection fraction, remote myocardium showed a decreased wall thickening suggesting relief of compensatory contractile work. This may be of value in the clinical assessment of regional function and suggests that evaluation of the coronary revascularization procedure with regards to regional function should be performed first 6 months or later after the intervention in patients with previous infarction. Further studies are warranted to elucidate the pathophysiological mechanism behind these findings.

## Abbreviations

CABG: coronary artery bypass surgery; CIHD: chronic ischemic heart; DE: delayed enhancement; ECG: electrocardiogram; FDG: ^18^F-2-deoxyglucose; Gd-DTPA: gadolinium diethylene triamine pentaacetic acid; IQR: interquartile range; MRI: magnetic resonance imaging; PCI: percutaneous coronary intervention; SPECT: single photon emission computed tomography; Tc: technetium.

## Competing interests

The authors declare that they have no competing interests.

## Authors' contributions

MU participated in design, performed data acquisition and image analysis, data analysis and wrote the manuscript. PC participated in design, data analysis, and writing the manuscript. JP and PJ participated in design and revised the manuscript for important intellectual content. HA conceived of the study, designed the study and participated in data analysis and writing the manuscript. All authors read and approved the final manuscript.

## Pre-publication history

The pre-publication history for this paper can be accessed here:

http://www.biomedcentral.com/1471-2261/10/4/prepub
